# Role of Ceramidases in Sphingolipid Metabolism and Human Diseases

**DOI:** 10.3390/cells8121573

**Published:** 2019-12-04

**Authors:** Farzana Parveen, Daniel Bender, Shi-Hui Law, Vineet Kumar Mishra, Chih-Chieh Chen, Liang-Yin Ke

**Affiliations:** 1 Department of Medical Laboratory Science and Biotechnology, College of Health Sciences, Kaohsiung Medical University, Kaohsiung 80708, Taiwan; fparveen.jh@gmail.com (F.P.); shlaw_0909@hotmail.com (S.-H.L.); vineetkmishra.jh@gmail.com (V.K.M.); 2 Institute of Biochemistry, Department of Chemistry, University of Cologne, 50674 Köln, Germany; dbender2@gmx.de; 3 Institute of Medical Science and Technology, National Sun Yat-sen University, Kaohsiung 80424, Taiwan; chieh@imst.nsysu.edu.tw; 4 Center for Lipid Biosciences, Kaohsiung Medical University Hospital, Kaohsiung 80708, Taiwan; 5 Graduate Institute of Medicine, College of Medicine & Drug Development and Value Creation Research Center, Kaohsiung Medical University, Kaohsiung 80708, Taiwan

**Keywords:** neutral ceramidase, ceramide accumulation, metabolic syndrome, insulin resistance, type 2 diabetes, Alzheimer’s disease, inflammatory bowel disease

## Abstract

Human pathologies such as Alzheimer’s disease, type 2 diabetes-induced insulin resistance, cancer, and cardiovascular diseases have altered lipid homeostasis. Among these imbalanced lipids, the bioactive sphingolipids ceramide and sphingosine-1 phosphate (S1P) are pivotal in the pathophysiology of these diseases. Several enzymes within the sphingolipid pathway contribute to the homeostasis of ceramide and S1P. Ceramidase is key in the degradation of ceramide into sphingosine and free fatty acids. In humans, five different ceramidases are known—acid ceramidase, neutral ceramidase, and alkaline ceramidase 1, 2, and 3—which are encoded by five different genes (*ASAH1*, *ASAH2*, *ACER1*, *ACER2*, and *ACER3*, respectively). Notably, the neutral ceramidase *N*-acylsphingosine amidohydrolase 2 (ASAH2) shows considerable differences between humans and animals in terms of tissue expression levels. Besides, the subcellular localization of ASAH2 remains controversial. In this review, we sum up the results obtained for identifying gene divergence, structure, subcellular localization, and manipulating factors and address the role of ASAH2 along with other ceramidases in human diseases.

## 1. Introduction

Ceramide and sphingosine-1 phosphate (S1P) are bioactive lipids of the sphingolipid pathway and play essential roles in cell signaling. In physical conditions, they play contrasting roles within cellular metabolism. Ceramide has been shown to be involved in stress-related cellular responses and apoptosis, whereas S1P stimulates cell survival, proliferation, and tissue regeneration [1,2,3,4]. Hence, maintaining the balance between ceramide and S1P is crucial for cells, as these bioactive lipids substantially contribute to cell fate decisions [5,6]. Because of their implications for cellular mechanisms, alterations in ceramide levels have been recognized in pathological conditions such as Alzheimer’s disease [7], type 2 diabetes [8], cardiovascular diseases [9], inflammatory bowel disease [10], Farber disease [11], sepsis, and colon cancer [12]. Cellular ceramide can be generated through three major metabolic pathways: through the hydrolysis of sphingomyelin through the actions of sphingomyelinases (also known as hydrolytic or SMase pathways), through the synthesis of dihydroceramide desaturase in a de novo pathway, or through ceramide synthase in a salvage pathway [13,14,15]. Ceramide catabolism is controlled by ceramidases that degrade ceramide into free fatty acids and sphingosine, which can be further phosphorylated by sphingosine kinase and which can produce sphingosine-1 phosphate (S1P). In high-fat diet (HFD)-fed mice, ceramide levels were pathologically high. With the transgenic expression of ceramidase in hepatic and adipose tissue, HFD-fed mice showed reduced systemic ceramide levels, improved insulin sensitivity, and hepatic steatosis [16]. In another study, the overexpression of sphingosine kinase 1 ameliorated insulin resistance in mice fed with a HFD [17]. These studies imply that either decreasing the level of ceramide or increasing the S1P level in HFD mice improves insulin sensitivity.

**Figure 1:**
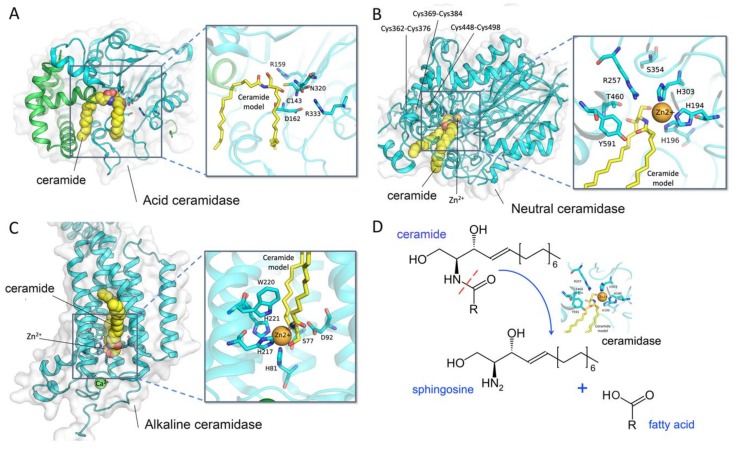
Crystal structure and the catalytic site of ceramidases. (**A**) Acid ceramidase (PDB ID: 6MHM); (**B**) neutral ceramidase (PDB ID: 4WGK); (**C**) alkaline ceramidase (PDB ID: 6G7O); and (**D**) the hydrolysis reaction of ceramidases. Docked ceramides are shown as yellow spheres and sticks. The structural representations were generated by PyMOL.

Ceramide is hydrolyzed by three types of ceramidases, which are differentially localized and are categorized according to their catalytic pH optimum: (i) acid ceramidase (Figure 1A), (ii) neutral ceramidase (Figure 1B), and (iii) alkaline ceramidase (Figure 1C). In this review, we summarize recent findings regarding ceramidases. In particular, the essential role of neutral ceramidase and its implications for human disease will be discussed. 

## 2. General Features of Ceramidases 

### 2.1. Nomenclature and Gene Loci

Ceramidases (EC3.5.1.23) cleave the *N*-acyl linkage of ceramide to produce sphingosine and free fatty acid (Figure 1D). In humans, five different genes encode for ceramidases. The gene *ASAH1* encodes for acid ceramidase *N*-acylsphingosine amidohydrolase 1 (ASAH1), which localizes to the p22 arm of chromosome 8. Neutral ceramidase *N*-acylsphingosine amidohydrolase 2 (ASAH2) is encoded by a gene located at q11.23 of chromosome 10. Finally, the genes for alkaline ceramidases ACER1, ACER2, and ACER3 are located at p13.3 of chromosome 19, p22.1 of chromosome 9, and q13.5 of chromosome 11, respectively (Table 1) [18].

### 2.2. Acid Ceramidases

Acid ceramidase ASAH1 is a 50-kDa protein, and the pH for its activity is 4.2–4.3 [19]. It is ubiquitously expressed and localizes to the lysosome to maintain intralysosomal ceramide homeostasis (Table 1) [20]. As a membrane protein, its secretion is very low [21]. It is very interesting to know that lysosomal targeting is mannose-6 phosphate receptor-dependent [22]. The *K*_M_ value of ASAH1 is about 389 to 413 µM, as is shown for ^14^C-labeled and BODIPY-conjugated substrate *N*-lauroylsphingosine (Table 2) [23]. In lysosomes, the activity of ASAH1 is dependent on a small protein called saposin D [24]. Saposin D deficiency results in tissue ceramide accumulation, suggesting that saposin D is a positive modulator of ASAH1. ASAH1 hydrolyzes mainly unsaturated ceramides containing C6–C16 acyl chains, in which the resulting sphingosine is used to generate S1P through sphingosine kinase [18]. The altered function of mutated ASAH1 has been recognized in spinal muscular atrophy with progressive myoclonic epilepsy [25] and Farber’s lipogranulomatosis [11]. In the case of metabolic disorders, there is either an alteration in acid ceramidase activity or a change in the expression level of ASAH1 in Alzheimer’s disease [26], cancer [27,28,29], and type 2 diabetes [30]. 

### 2.3. Neutral Ceramidases

Neutral ceramidase ASAH2 is 85.5 kDa in size. Initially, ASAH2 was characterized as a mitochondria protein when overexpressed in HEK293 cells [31,32], but later it was also identified as a membrane protein with *O*-glycosylation (Table 1) [33,34]. Its detailed mechanisms remain unclear. The *K*_M_ value of neutral ceramidase is about 60.1 µM, using *D*-erythro-C12-4nitrobenzo-2-oxa-1,3-diazole-ceramide as the substrate (Table 2) [35]. Neutral ceramidase is only expressed at the intestinal brush border of the small intestine: it has a role in digesting dietary sphingolipid to regulate the sphingolipid balance in the human gut [33]. 

### 2.4. Alkaline Ceramidases

Alkaline ceramidase ACER1 has a mass of approximately 31 kDa and is 264 amino acids in length. It has been reported to be a membrane protein localized at the ER [36,37] with a catalytic pH optimum of 9.0 (Table 1) [38]. ACER1 is highly expressed in the differentiated interfollicular epidermis, sebaceous gland, and infundibulum. ACER1-deficient mice showed increased transepidermal water loss and aberrant hair shafts. In contrast, the upregulation of ACER1 through Ca^2+^ influx induced growth arrest and the differentiation of human epidermal keratinocytes. Therefore, ACER1 is believed to be a key regulator of keratinocyte differentiation and has a fundamental role in skin barrier formation [36,37]. 

ACER2 is a 31-kDa membrane protein of 275 amino acids. Seven putative transmembrane domains allow for protein association with the Golgi apparatus [39] (Table 1). The *K*_M_ value for the ceramide of ACER2 is about 94.8~98.5 µM depending on the substrate derivative used (Table 2) [40]. ACER2 is expressed in the placenta, pancreas, and heart [39]. ACER2 expression is regulated by the tumor suppressor p53 and hypoxia-inducible factor 2α [41,42,43,44]. Hence, ACER2 mRNA expression has been found to be increased in human cancer tissue in the liver and colon when compared to samples of healthy individuals [39]. In various stress conditions, such as in the presence of glucocorticoid or reactive oxygen species (ROS), cellular ACER2-produced sphingosine induces programmed cell death by increasing the production of reactive oxygen species (ROS) in response to DNA damage [41]. 

ACER3 is a 267-amino-acid membrane protein of approximately 32.6 kDa. It is ubiquitously expressed, with the highest expression levels found in human placental tissue [45]. Localized in both the ER and Golgi complex, it catalyzes the hydrolysis of unsaturated long acyl chains (Table 1). ACER3 has been recognized to regulate cell proliferation and apoptosis [45,46]. Knockout mice have shown impairment in motor coordination and premature neurodegeneration, which suggests ACER3 has a protective role in brain function [47].

## 3. Physiology of Neutral Ceramidase ASAH2

### 3.1. Tissue Distribution Levels of Neutral Ceramidases in Rats and Mices

**Figure 2:**
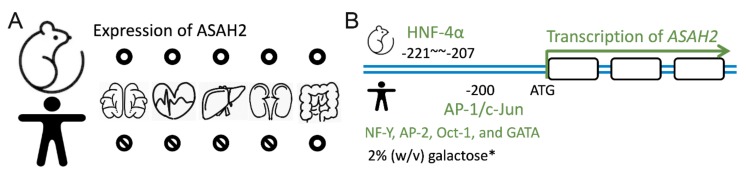
Tissue expression of ASAH2 and transcriptional factors stimulating expression. (**A**) ASAH2 is expressed in the brain, heart, liver, kidneys, gut, and small intestine of mice (upper panel). In contrast, it is only expressed in the gut and small intestine of humans (lower panel). (**B**) The transcriptional factor hepatocyte nuclear factor 4α (HNF-4α) binds to −207 to −221 of *ASAH2* in mice (NM_018830; *Mus musculus*). In contrast, together with c-Jun, the transcriptional factor AP-1 binds to −200 and regulates human *ASAH2* transcription (NM_019893; *Homo sapiens*). Other transcriptional factors such as NF-Y, AP-2, Oct-1, and GATA have been reported as regulating the expression of human *ASAH2*. Note that in a yeast-expressing system, 2% (*w*/*v*) galactose also enhances expression.

Neutral ceramidase ASAH2 was first purified from rat brains by Tani et al. in 2000 [58]. In 2003, they demonstrated the role of *O*-glycosylation in the tissue localization of ASAH2 in the mucin box. *O*-glycosylated protein was found to be 112 kDa in size in mouse kidneys, which localized to the plasma membrane. In contrast, a soluble ASAH2 isoform was found in mouse livers that lacked *O*-glycan modification [34]. The expression of ASAH2 was detected in various tissues, including the small intestines, colons, livers, kidneys, brains, and hearts of mice (Figure 2A, upper panel) [33]. There is evidence that it is expressed in the apical membranes of collecting ducts, the proximal and distal tubes of the kidney, as well as in the endosome-like structure of hepatocytes [59]. 

### 3.2. Human Expression of Neutral Ceramidases

Human ASAH2 was first identified in the small intestine (Figure 2A, lower panel) [33,60,61]. In human embryonic kidney cells, which exogenously overexpress ASAH2, the protein was found to localize to mitochondria [31]. In contrast to this finding, Hwang and coworkers showed that ASAH2 localizes to the plasma membrane [32] and is highly glycosylated with *O*-glycans [34]. In line with that, Tani and coworkers showed that truncation or mutation of the C-terminal part of ASAH2 retains the protein in the ER while simultaneously abrogating ASAH2 activity [62]. Sakamoto and coworkers found expressed ASAH2 in colon cancer cells. There, ASAH2 localized to the Golgi as well as to the plasma membrane. Moreover, specific ASAH2 activity was recognized in the Golgi, as evidenced by ceramide turnover and inhibited C6-ceramide-induced cell death and Golgi fragmentation [54]. In contrast to mice, *ASAH2* is not expressed at a significant level in the human brain. Instead, a partial duplication of *ASAH2* on chromosome 10q11.23 (called *ASAH2L*) is abundantly expressed in brain tissue. Most interestingly, its expression has been recognized to decrease in correlation with age and late-onset of Alzheimer’s disease [50].

### 3.3. Factors Manipulating ASAH2 Expression and Activity

Using a genome-wide study, Maltesen el al. predicted that the hepatocyte nuclear factor 4α (HNF-4α) binding site is at position −207 to −221 of *ASAH2* (NM_018830; *Mus musculus*) (Figure 2B, upper panel). Using a supershift analysis and HNF-4α overexpression and knockdown experiments, the authors confirmed the role of HNF-4α in controlling ASAH2 expression and therefore lipid metabolism [63]. In human cells, transcription factor AP-1 recognizes the −200-bp promoter region upstream of *ASAH2*. Acting together with c-Jun, AP-1 regulates serum-induced human *ASAH2* gene transcription (Figure 2B, lower panel) [64]. Due to a transcriptional response element, other transcription factors, including nuclear transcription factor Y (NF-Y), AP-2, Oct-1, and GATA, also regulate the transcriptional activity of human *ASAH2* [65]. In a yeast expression system, Galadari and coworkers showed that the expression of ASAH2 could be induced by 2% (*w*/*v*) galactose in a synthetic-complete medium (SC-Ura−) [35]. The downregulation of ASAH2 expression could be achieved through gemcitabine or serum starvation. Moreover, Na^+^, Ca^2+^, Mg^2+^, and Mn^2+^ increased the enzyme activity of purified yeast microsomal ASAH2. In contrast, Zn^2+^, Cu^2+^, and Fe^2+^ inhibited ASAH2 activity [35].

### 3.4. The Amino Acid Sequence of ASAH2

**Figure 3:**
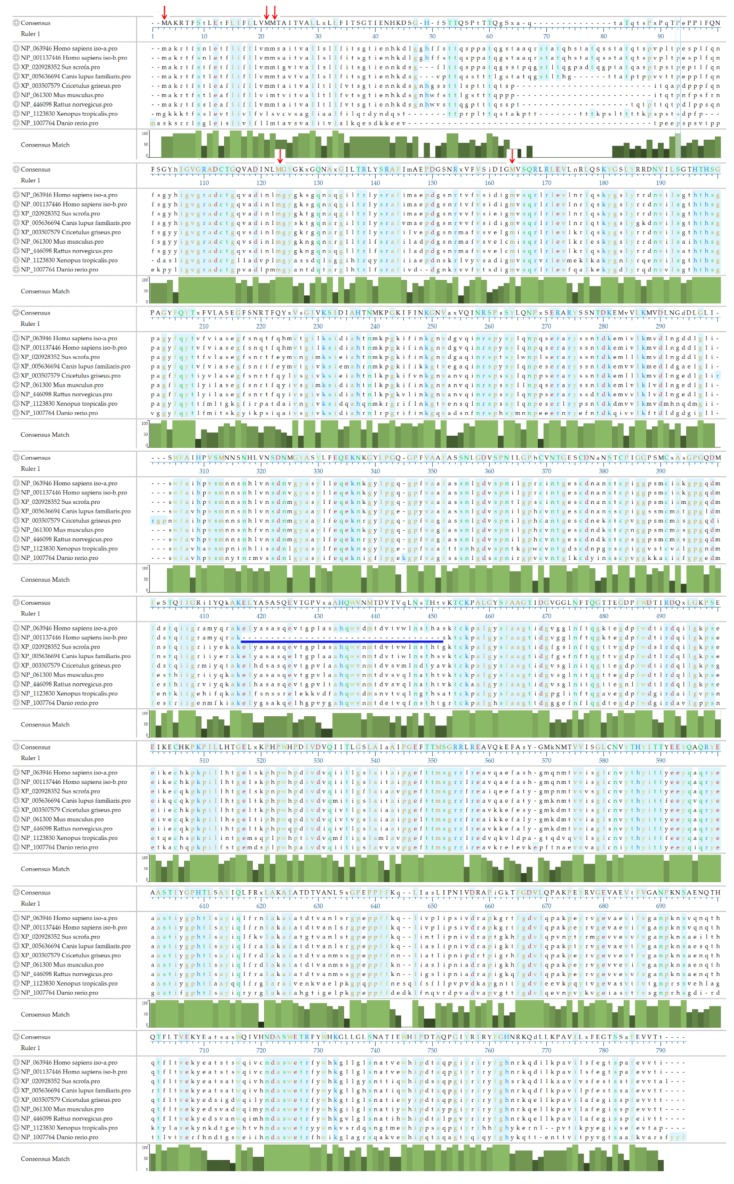
Alignment of ASAH2 amino acid sequences from different species using the Clustal W method. NP_063946 ASAH2 isoform 1 from *Homo sapiens*; NP_001137446 ASAH2 isoform 2 from *Homo sapiens*, with alternative splicing skipping amino acids 410–444; XP_020928352 ASAH2 from *Sus scrofa* (pigs); XP_005636694 ASAH2 from *Canis lupus familiaris* (dogs); XP_003507579 ASAH2 from *Cricetulus grisesu* (hamsters); NP_061300 ASAH2 from *Mus musculus* (mice); NP_446098 ASAH2 from *Rattus norvegicus* (rats); NP_1123830 from *Xenopus tropicalis*; NP_1007764 from *Danio rerio*. Amino acids are colored according to different chemical properties. Identical amino acid residues are shown with a blue background. Note that some issues are listed below: The alternative splicing of human ASAH2 leads to a truncation of isoform 2, which lacks 35 amino acids from position 410 to 444 (blue line). The detailed mechanisms are not clear. Position 34 to 103 is the most variable region of ASAH2. It may lead to enormous differences between the subcellular location, function, and expression levels in humans and other animals. The N-terminal type 2 membrane signal-anchor peptide FLIFLLVMMXXX (a.a.12 to 33) and the amidase motif NLGDVSPNXLGPXC (a.a.353 to 366) are relatively conserved, and their functions have been well studied. Red arrows indicate multiple putative start codons in the N-terminal of ASAH2. These might lead to various ASAH2 isoforms that differ in length and in terms of their intracellular localization.

Two isoforms of human neutral ceramidases have been identified. ASAH2 isoform 1 (NP_063946; *Homo sapiens*) is 780 amino acids in length, while ASAH2 isoform 2 (NP_001137446; *Homo sapiens*) comprises only 745 amino acids. Alternative splicing leads to the truncation of isoform 2, which lacks 35 amino acids, from position 410 to 444 (Figure 3, blue line). The occurrence and the functional differences between the two isoforms are still not clear. An alignment of *ASAH2* orthologs from humans, pigs (*Sus scrofa*; XP_020928352), dogs (*Canis lupus familiaris* XP_005636694), hamsters (*Cricetulus grisesu*; XP_003507579), mice (*Mus musculus*; NP_061300), rats (*Rattus norvegicus*; NP_446098), frogs (*Xenopus tropicalis*; NP_1123830), and zebrafish (*Danio rerio*; NP_1007764) revealed that the most variable region ranges from position 34 to 103, which includes hypothetical *O*-glycosylation sites ranging from amino acids 62 to 98. Besides, in hamsters, mice, rats, frogs, and zebrafish, some of the nucleotide sequences of *ASAH2* are missing (Figure 3). We surmise that the variable region of ASAH2 may lead to huge differences between the subcellular location, function, and expression levels in humans and other animals. The *N*-terminal type 2 membrane signal-anchor peptide FLIFLLVMMXXX (a.a.12 to 33) and the amidase motif NLGDVSPNXLGPXC (a.a.353 to 366) are relatively conserved, and their functions have been well studied. However, there are multiple putative start codons in the N-terminal of *ASAH2* mRNA. These might lead to various ASAH2 isoforms that differ in the length of their N-terminus and might significantly influence intracellular localization of the ASAH2 polypeptide. 

### 3.5. Structure of Human ASAH2

The X-ray crystal structure of ASAH2 was described by Airola and coworkers in 2015 [57]. They overexpressed the extracellular domain (amino acid residues 99 to 780) of human ASAH2 in an insect Sf9 cell line and showed enzyme activity (*K_M_* = 33.41 uM, *Kcat* = 61.93 min^−1^). In the structure, a narrow hydrophobic substrate-binding pocket was identified. They suggested that His196, Arg257, Tyr579, and Tyr591 in the binding pocket of human ASAH2 play critical roles in catalyzing the hydrolysis of ceramide (Figure 1B). The previously identified active site Ser354 is located at the base of the pocket to stabilize the position of Arg257. Disulfide bonds are present in the catalytic domain between Cys362 and Cys376, Cys369 and Cys384, and Cys448 and Cys498, indicating a likely explanation for sensing the reducing agents. Besides, N-linked glycans can be observed at Asn151, Asn217, Asn308, and Asn440 in the catalytic domain and at Asn730 in the Ig-like domain. Through superposition of the bacterial counterpart to ASAH2, the authors observed that human ASAH2 has an additional 30-residue subdomain replacement (Leu358 to Lys387) and a differently positioned η2 loop α8 element (Ala451 to Ile487) [57,66].

## 4. Role of Ceramidases in Pathological Conditions 

### 4.1. Genetic Disorders Related to Ceramidases

#### 4.1.1. Genetic Variations of *ASAH1*

Farber disease is a rare and severe autosomal genetic disorder leading to the intralysosomal accumulation of ceramide in various tissues. It is caused by the lack of acid ceramidase ASAH1 or by a reduction in its activity due to missense mutations [11,67,68]. Phenotypically, Farber disease is characterized by hepatomegaly and alterations in neurological function [69]. *ASAH1* knockout in mice is embryonically lethal, while human patients live for up to 2 years [70,71]. It can be concluded that this gene is necessary for survival and essential for systemic sphingolipid metabolism. 

#### 4.1.2. Genetic Variations of *ASAH2*

The genetic mutations of *ASAH2*, which cause a phenotype in humans, are still unknown. However, in the model plant *Arabidopsis thaliana*, mutations in the ASAH2 ortholog *At*NCER1 cause the accumulation of hydroxyceramide. It is for this reason that these plants are more susceptible to oxidative stress. Underlining this, plants overexpressing *At*NCER1 show higher oxidative stress tolerance, which illustrates the protective role of ASAH2 toward stress [72]. From this, the role of human ASAH2 in the regulation of ROS-induced cell stress responses might be assumed.

#### 4.1.3. Genetic Variations of *ACER3*

Progressive leukodystrophy is a rare genetic disorder characterized by the abnormal production of myelin, which affects the central nervous system [55]. In 2016, Edverdson and coworkers investigated the genetic background of leukodystrophy in a patient through exome sequencing and found that the patient was homozygous for a p.E33G mutation in alkaline ceramidase ACER3. Due to the defect in ACER3, long-chain ceramides and dihydroceramide accumulated in the plasma and brain of the leukodystrophy patient [56]. Through crystal structure and computational studies, Vasiliauskaité-Brooks et al. uncovered that E33 is a Ca^2+^ binding site for ACER3 enzymatic function [73]. 

### 4.2. Metabolic Disorders

**Figure 4:**
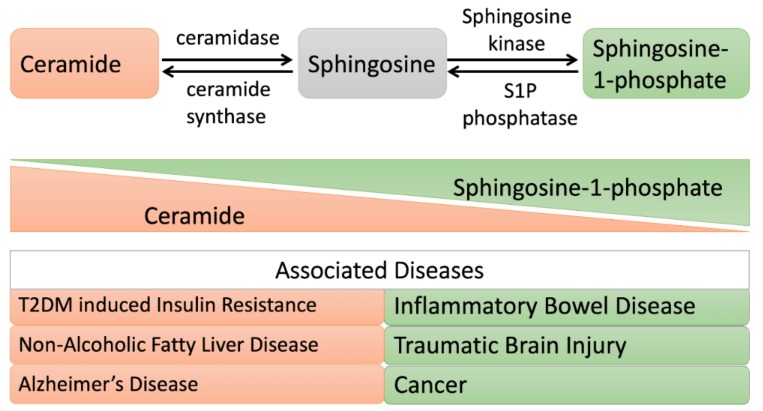
Altered ceramide/sphingosine-1 phosphate balance during metabolic disorders and shifting cell fate toward apoptosis and proliferation, respectively. When the ceramide level is elevated: cells undergo apoptosis. A high level of ceramide is observed in patients with insulin resistance, Alzheimer’s disease, or nonalcoholic fatty liver disease (NAFLD). While, when sphingosine-1 phosphate (S1P) is elevated: cells undergo a proliferative mode. A high level of S1P is observed in patients with inflammatory bowel disease, traumatic brain injury, and cancer.

An oversupply of fat results in metabolic dysregulation, leading to obesity, insulin resistance, nonalcoholic fatty liver disease (NAFLD), and potentially Alzheimer’s disease [74,75,76]. Among the lipids involved in these metabolic diseases, diacylglycerol (DAG) and ceramide are the most studied candidates (Figure 4).

#### 4.2.1. Insulin Resistance

Obesity cases show a compromised insulin sensitivity due to lipid oversupply and lipid-induced oxidative stress [77,78,79]. The increased bioactive lipid in obesity, ceramide, contributes to insulin resistance and lipotoxicity. The quantification of plasma ceramide as a biomarker could predict major adverse cardiac events [80,81,82,83]. Zhu and colleagues showed that ASAH2 treatment might overcome palmitic acid (PA)-induced insulin resistance and also block PA-induced oxidative stress where ceramide is involved (Table 3) [84,85]. Xie et al. also demonstrated that ceramide depletion through the overexpression of acid ceramidase either in the liver or in adipose tissue ameliorates the ceramide-activated protein kinase C isoform PKCζ and therefore improves hepatic steatosis and insulin sensitivity [16]. Furthermore, Chavez et al. showed that long-chain saturated FFAs suppress the insulin stimulation of glucose uptake regulator AKT/protein kinase B and promote the synthesis of ceramide and sphingosine: they have also been shown to inhibit insulin action [86]. To study the contribution of ceramide and sphingosine to the inhibition of insulin resistance mediated by FFAs, the same group showed that the overexpression of acid ceramidase in C2C12 myotubes stimulated the abnormal accumulation of ceramide, describing its role in muscle insulin resistance [30].

#### 4.2.2. Cardiovascular Disease 

Adiponectin is the most abundant hormone secreted by adipocytes. It is involved in regulating glucose metabolism and fatty acid breakdown. Its reduction plays a very significant role in obesity-linked diseases, including insulin resistance, T2DM, and cardiovascular diseases [89,90]. Adiponectin has a protective role in cardiovascular diseases because it reduces oxidative stress and prevents endothelial cell apoptosis [91,92]. Upon adiponectin treatment, ASAH2 activity increases, while simultaneously, tumor necrosis factor-α (TNF-α)-induced oxidative stress is reduced in human umbilical vein endothelial cells (HUVECs). The interaction between Caveolin 1 (Cav1) and ASAH2 increases upon adiponectin treatment, which is the inhibiting factor of TNF-α-induced vascular inflammation [87]. AdipoR1 and AdipoR2 are adiponectin receptors [93]. The pleiotropic actions of adiponectin, which are linked to the ceramide signaling pathway, activate adiponectin receptors and lower ceramide levels by activating its ceramidase activity [94]. ADIPOR1 and ADIPOR2 possess intrinsic basal ceramidase activity enhanced by adiponectin; however, the ceramidase activity is low. In that study, the authors found that the side chains of residues are coordinated with zinc, which promotes zinc-bound hydroxide ions for nucleophilic attacks on the ceramide amide carbonyl [95]. However, further work is required to explore ceramidase activity and substrate specificity for these receptors.

#### 4.2.3. Alzheimer’s Disease

Alzheimer’s disease is a neurodegenerative disorder characterized by amyloid-beta (Aβ) accumulation and phosphorylated-tau protein deposition [96]. Many factors can be responsible for the aberrant apoptosis of neuronal cells. In this context, ceramide has a significant role in neuron apoptosis induction, which may be the possible culprit in this neuropathological disorder [97,98]. In 2010, He et al. found an increased level of acid sphingomyelinase and acid ceramidase, which results in a reduction of sphingomyelin and an increase in ceramide levels [88]. The increasing ceramide further stabilizes APP-cleaving enzyme 1 (BACE1) and promotes the overproduction of Aβ [99,100]. 

#### 4.2.4. Traumatic Brain Injury

Traumatic brain injury (TBI) is defined as a sudden mechanical injury to the brain that progresses in terms of neuronal degeneration, followed by the disruption of synaptic circuits [101]. Increased levels of sphingomyelin and sphingosine have been recognized in traumatic brain injury, which suggests the dysregulation of sphingolipid metabolism [102]. Further investigation has revealed the intracellular accumulation of sphingosine in mitochondria due to increased ASAH2 activity and decreased sphingosine kinase 2 activity (in TBI cases). It has also been mentioned that sphingosine accumulation causes decreased cytochrome oxidase activity, a crucial enzyme in the electron transport chain of mitochondria. The knockdown of ASAH2 partially improves TBI mouse brain function [51].

#### 4.2.5. Cancer

ASAH2, which is highly expressed in the small intestine along the brush border, plays an essential role in the pathogenesis of colorectal cancer. Garcia-Barros et al. demonstrated that the inhibition of ASAH2 elevates the ceramide level (with an increase in apoptosis and autophagy) and also leads to the suppression of the components responsible for colon cancer development, β-catenin, and ERK. Additionally, ASAH2 inhibition induced delayed tumor growth in a xenograft model with an increased ceramide level and decreased proliferation, which confirmed the role of neutral ceramidase in the regulation and development of colon cancer [103]. In a study by Coant et al., it was reported that the dephosphorylation of GSK3β is activated upon the inhibition of ASAH2 and hence is needed for β-catenin phosphorylation and degradation. In their study, AKT was a crucial target for growth, suppressing the role of ceramide. The AKT pathway requires the presence of ASAH2 for its basal level of activity: this was revealed through the inhibition of ASAH2 in colorectal cells, which induces a significant reduction in the phosphorylation of GSK3β and activates the kinase. It was also reported that this inhibition causes the dephosphorylation and incompetency of AKT. This came to light through the overexpression of AKT (constitutively active AKT-DD, phospho-mimic), which leads this mutant to overcome the growth-suppressive effects of ASAH2 inhibition in colorectal cancer (CRC) cells. Additionally, Coant et al. also demonstrated that the inhibition of ASAH2 induces growth delay in the case of xenograft tumors taken from CRC cell lines. It was interesting to note that xenograft tumors from consecutively active AKT cells become resistant to ASAH2 inhibition. Hence, ceramide may play with the attenuation of AKT activation, and also ASAH2 is a key mediator in maintaining ceramide functions. Thus, this explains the role of ASAH2 in regulating the basal activation of AKT, and therefore ASAH2 can be proposed as a novel target for colon cancer therapy [52]. 

#### 4.2.6. Inflammatory Bowel Disease

Inflammatory bowel disease (IBD) is defined as chronic inflammation of the intestinal tract due to abnormal mucosal immune responses. Affected individuals experience abdominal symptoms, including diarrhea, abdominal pain, vomiting, and bloody stool [104]. In IBD, ceramide accumulates in microdomains of cholesterol- and sphingolipid-enriched membranes, resulting in impairment of the barrier function of the gut [105]. ASAH2 is primarily expressed on the brush border of the small intestine. It has been found that lost in its enzymatic activity or the knockout of ASAH2 decreases the level of sphingosine [33]. However, in 2012, Snider and coworkers showed that under inflammatory conditions in the intestinal epithelium, ASAH2 is not the ceramidase responsible for sphingosine generation. In ASAH2−/− mice (following dextran sulfate sodium to induce IBD), the loss of ASAH2 resulted in an elevated level of S1P and increased systemic inflammation. This study suggested that ASAH2 might play an essential role in membrane maintenance and in the barrier function to protect against commensal bacteria-induced inflammation [53]. As was previously mentioned in this review, the adiponectin receptor shows ceramidase activity and is stimulated by adiponectin or palmitate in beta cells. This activity can contribute to the downstream generation of S1P [94] and to protection from apoptosis upon the knockout of ASAH2 [53]. Colitis is a major group of IBDs characterized by chronic mucosal inflammation [106]. IBD leads to colitis-associated cancer (CAC) and colorectal cancer [107]. To further demonstrate the role of ceramidase in IBD-associated cancer, Espaillat and coworkers showed an elevation in the expression of acid ceramidase in a mice model of colitis (induced by dextran sulfate sodium) [108]. Using an intestinal epithelial injury model, their work revealed that the loss of acid ceramidase in myeloid cells inhibits tumorigenesis through the impairment of neutrophil recruitment to the colon mucosa due to the loss of chemokine and cytokine production, which protects against colitis-associated cancer (CAC) [108].

## 5. Ceramidase Inhibitors

In diseases such as cancer, the overexpression of ceramidase results in the excessive generation of sphingosine-1 phosphate, which leads to larger tumor growth and more resistance to chemotherapy. Recently, targeting ASAH1 for cancer treatment has been shown to enhance the efficacy of chemotherapy, which identifies ASAH1 as a therapeutic target. Thus, several ASAH1 inhibitors have been developed for use in cancer therapies [109,110]. The well-known inhibitors of ceramidases are B-13, D-*e*-MAPP, and NOE [111]. Among these, B-13 has poor potency toward acid ceramidase, but a derivative of B-13 is LCL-464, which possesses high potency toward acid ceramidase [112]. Further, the structural modification of B-13 and LCL-464 generates more potent inhibitors against ASAH1 and ASAH2. These inhibitors have been shown to enhance the level of ceramide, thus inducing apoptosis in breast cancer cell lines (MDA-MB-231) [113]. The inhibition of ASAH1 also blocks angiogenesis via a pathway that is independent of VEGF [114]. For example, ASAH1 is a critical regulator in the case of prostate cancer progression, and when it is suppressed by the knocking down of ASAH1 using siRNA, this results in the amelioration of tumor growth and sensitization toward chemotherapy [115]. Recently, ASAH1 was recognized as a de novo glioblastoma drug target. Doan et al. identified Carmofur as an ASAH1 inhibitor that can cross the blood–brain barrier. It is highly effective and targets glioblastoma cancer stem cells [116]. Carmofur is an approved drug against colorectal cancer in Japan [117]. Therefore, this work suggests that the inhibition of acid ceramidases elevates ASAH1 levels, induces apoptosis, blocks angiogenesis, and improves the response to chemotherapy.

## 6. Conclusions

Ceramide plays a crucial role in metabolic and disease states, and therefore, the degrading enzyme ceramidase is the critical regulator that maintains ceramide homeostasis inside cells. A disturbance in the balance between ceramide and sphingosine-1 phosphate interferes with healthy metabolism. Consumption of a high-fat diet leads to the development of obesity-related diseases, where ceramide is involved. An accumulation of ceramide has been observed in NAFLD, T2DM, cancer, IBD, and AD. Although ASAH2 may improve insulin sensitivity and hepatic steatosis and inhibits TNF-α-induced vascular inflammation, only a few studies have targeted the manipulation of ASAH2 expression as a therapeutic approach, as ASAH2 distribution and its functions remain unclear in many respects. In summary, finding a suitable animal model and gaining a better understanding of ASAH2 will be of critical importance for clarifying its role in human diseases and in terms of its potential use in the treatment of metabolic disorders and neurodegenerative diseases.

## Figures and Tables

**Table 1:** Expression and subcellular localization of human ceramidases.

Ceramidase	Encoded by Gene	Optimal pH	Expression	Subcellular Localization	Associated Diseases
Acid ceramidase	*ASAH1,* located at p22 of chromosome 8	∼4.0–4.5	Heart, kidneys, lungs, placenta, etc. [48]	Lysosomes [48]	Farber’s disease, Alzheimer’s disease, cancer, diabetes, and spinal muscular atrophy [11,25,26,27,28,30,49]
Neutral ceramidase	*ASAH2,* located at q11.23 of chromosome 10	∼7.0–7.4	Small intestine [33]	Mitochondria (HEK293T overexpressing cells) [31]	Alzheimer’s disease [50], various metabolic diseases [51,52,53]
				Plasma membrane (HEK293T overexpressing cells) [32,34]	
				Mitochondria and Golgi (HCT116 overexpressing cells) [54]	
Alkaline ceramidase	*ACER1*, located at p13.3 of chromosome 19	∼9.0	Skin	ER	Progressive leukodystrophy [55,56]
	*ACER2*, located at p22.1 of chromosome 9		Placental tissue	Golgi complex [39,40]	
	*ACER3*, located at q13.5 of chromosome 11		Ubiquitous, highly expressed in placental tissue	ER and Golgi complex [45,46]	

ASAH1, *N*-acylsphingosine amidohydrolase 1; ASAH2, *N*-acylsphingosine amidohydrolase 2; ACER1, alkaline ceramidase 1; ACER2, alkaline ceramidase 2; ACER3, alkaline ceramidase 3; ER, endoplasmic reticulum; Golgi, Golgi apparatus.

**Table 2:** *K*_M_ values of ceramidases.

Name	pH	Temp.	*K_M_* (µM)	*Vmax* (nmol/min mg)	Substrate	Note	References
Acid ceramidase	4.5	37	389	462.97	*N*-lauroylsphingosine	pH 4.5, 37 °C, recombinant ceramidase expressed in CHO cells, 14C-labeled substrate	[23]
			413.2	33.33		pH 4.5, 37 °C, recombinant ceramidase expressed in CHO cells, BODIPY-conjugated substrate	
Neutral ceramidase	7.5	37	60.1 33.41	0.68 N/A	*D*-erythro-C12-4nitrobenzo-2-oxa-1,3-diazole-ceramide NBD-C12-ceramide	pH 7.5, 37 °C, recombinant ceramidase 28 °C, recombinant enzyme (mutant, residues 99–780)	[35] [57]
Alkaline ceramidase (ACER2)*	9.0	37	98.5 94.8	0.0237 0.0261	C16:0-ceramide C18:0-ceramide	pH 9.0, 37 °C	[40]

CHO: Chinese hamster ovary; BODIPY, an abbreviation for boron-dipyrromethene fluorescent dye; ACER2: alkaline ceramidase 2; *: no *K_M_* value information for ACER1 or ACER3.

**Table 3:** Ceramidase and metabolic disorders.

Disease	ASAH2 Expression	Ceramide Level	S1P Level	Tissue/Cells	References
Insulin resistance	Decreased	Increased	Not known	INS-1 pancreatic β cells, H4IIEC3 hepatocytes	[84,85]
Cardiovascular diseases	Activity decreased	Not known	Not Known	Human umbilical vein endothelial cells (HUVECs)	[87]
Alzheimer’s disease	Not known	Ceramide accumulation	Decreased	Human brain	[88]
Traumatic brain injury	Increased activity	Decreased	No difference	Mouse brain, mitochondria dysfunction	[51]
Cancer	Increased expression	Not known	Not known	CaCo2BBe, HCT116 colon cancer cells	[52]
Inflammatory bowel diseases	Decreased activity	Increased in colon epithelium	Increased upon ASAH2 knockout	Gut from DSS-treated C57BL/6 mice	[53]

INS-1: insulin-secreting beta cell; DSS: dextran sulfate sodium.

## Abbreviations

S1P	Sphingosine-1 phosphate
AD	Alzheimer’s disease
ASAH1 ASAH2 ACER1 ACER2 ACER3	Acid ceramidase; *N*-acylsphingosine amidohydrolase 1 Neutral ceramidase; *N*-Acylsphingosine Amidohydrolase 2 Alkaline ceramidase 1 Alkaline ceramidase 2 Alkaline ceramidase 3
BODIPY CHO ER FFA IBD NAFLD T2DM	boron-dipyrromethene fluorescent dye Chinese hamster ovary Endoplasmic reticulum Free fatty acid Inflammatory bowel disease Nonalcoholic fatty liver disease Type 2 diabetes mellitus
TBI	Traumatic brain injury
